# Impact of diabetes on surgery and radiotherapy for breast cancer

**DOI:** 10.1007/s10549-023-06915-1

**Published:** 2023-03-30

**Authors:** Ross Lawrenson, Chunhuan Lao, James Stanley, Ian Campbell, Jeremy Krebs, Ineke Meredith, Jonathan Koea, Andrea Teng, Dianne Sika-Paotonu, Jeannine Stairmand, Jason Gurney

**Affiliations:** 1 Medical Research Centre, The University of Waikato, Hamilton, New Zealand; 2 Strategy and Funding, Waikato Hospital, Hamilton, New Zealand; 3 Department of Public Health, University of Otago, Wellington, New Zealand; 4 School of Medicine, The University of Auckland, Auckland, New Zealand; 5 General Surgery, Waikato Hospital, Hamilton, New Zealand; 6 Department of Medicine, University of Otago, Wellington, New Zealand; 7 General Surgery, Wakefield Hospital, Wellington, New Zealand; 8 General Surgery, Waitakere Hospital, Auckland, New Zealand; 9 Medical Surgery, The University of Auckland, Auckland, New Zealand; 10 Department of Pathology & Molecular Medicine, University of Otago, Wellington, New Zealand

**Keywords:** Diabetes, Breast cancer, Mastectomy, Breast conserving surgery, Breast reconstruction, Treatment delay, Radiotherapy

## Abstract

**Purposes:**

This study aims to examine whether diabetes has an impact on the use of surgery and adjuvant radiotherapy in treating women with localised breast cancer.

**Methods:**

Women diagnosed with stage I–III breast cancer between 2005 and 2020 were identified from Te Rēhita Mate Ūtaetae—Breast Cancer Foundation New Zealand National Register, with diabetes status determined using New Zealand’s Virtual Diabetes Register. The cancer treatments examined included breast conserving surgery (BCS), mastectomy, breast reconstruction after mastectomy, and adjuvant radiotherapy after BCS. Logistic regression modelling was used to estimate the adjusted odds ratio (OR) and 95% confidence interval (95% CI) of having cancer treatment and treatment delay (> 31 days) for patients with diabetes at the time of cancer diagnosis compared to patients without diabetes.

**Results:**

We identified 25,557 women diagnosed with stage I–III breast cancer in 2005–2020, including 2906 (11.4%) with diabetes. After adjustment for other factors, there was no significant difference overall in risk of women with diabetes having no surgery (OR 1.12, 95% CI 0.94–1.33), although for patients with stage I disease not having surgery was more likely (OR 1.45, 95% CI 1.05–2.00) in the diabetes group. Patients with diabetes were more likely to have their surgery delayed (adjusted OR of 1.16, 95% CI 1.05–1.27) and less likely to have reconstruction after mastectomy compared to the non-diabetes group—adjusted OR 0.54 (95% CI 0.35–0.84) for stage I cancer, 0.50 (95% CI 0.34–0.75) for stage II and 0.48 (95% CI 0.24–1.00) for stage III cancer.

**Conclusions:**

Diabetes is associated with a lower likelihood of receiving surgery and a greater delay to surgery. Women with diabetes are also less likely to have breast reconstruction after mastectomy. These differences need to be taken in to account when considering factors that may impact on the outcomes of women with diabetes especially for Māori, Pacific and Asian women.

## Introduction

Breast cancer is the most common cancer for New Zealand women and the third most common cancer overall, with around 3500 new cases per year [1]. Approximately 95% of patients diagnosed with breast cancer in New Zealand have stage I–III disease at initial diagnosis [2]. Most of these breast cancer patients will have surgery [3, 4]. Historically, surgical treatment of breast cancer has involved mastectomy, but since the 1980’s, breast conserving surgery (BCS) followed by radiotherapy to prevent local recurrence has been the recommended treatment for early stage breast cancer [3–5]. The use of mastectomy, reconstruction after mastectomy, BCS, and adjuvant radiotherapy may vary depending on patient, tumour and health system factors, such as patient’s age, comorbidities, ethnicity, cancer stage and public or private hospital [3, 4].

Diabetes is a significant comorbidity that can affect the treatment of breast cancer [6]. Around 15% of the population will be diagnosed with both diabetes and cancer in their lifetime [7]. There is some evidence that patients with diabetes are less likely to receive aggressive curative treatment for their cancer, and less likely to receive guideline-concordant care than patients without diabetes [8, 9]. A Canadian study showed that women with diabetes were slightly less likely to receive radiotherapy after BCS than women without diabetes, although the differences were no longer apparent after adjustment for other comorbidities [10]. Several factors may explain this phenomena, including clinical concern for treatment toxicity and effectiveness in patients with diabetes [9]. Overseas studies have shown that patients with diabetes had not only worse all-cause mortality than patients without diabetes but also worse breast cancer-specific mortality [11–14]. Therefore the differences in breast cancer treatment for patients with and without diabetes need to be taken in to account when exploring what factors may impact on breast cancer outcomes.

New Zealand has a publicly funded health system with all hospital care available to citizens and permanent residents. However, some women may choose to access care in the private sector. The breast cancer register capture information on all women whether they are treated in the public or private sector. There are concerns that some patient groups are treated differently especially in a New Zealand setting (our Māori and Pacific patients). As diabetes is more prevalent in Māori and Pacific, it is important to understand the impact comorbidities such as diabetes have in cancer treatment. This study aims to explore whether diabetes affects the treatment of stage I-III breast cancer and whether the impact is the same between different ethnic groups. It will use the comprehensive Te Rēhita Mate Ūtaetae—Breast Cancer Foundation New Zealand National Register (NBCR) data which has detailed information on breast cancer treatments, alongside the Virtual Diabetes Register (VDR) which provides a national prevalent cohort of patients with diabetes. We will examine whether diabetes has an impact on the use of surgical treatment, surgery type, use of reconstruction after mastectomy and adjuvant radiotherapy after BCS for stage I-III breast cancer, and examine whether there are differences between ethnic groups.

## Methods

Women diagnosed with stage I–III breast cancer between 2005 and 2020 were identified from the NBCR. Stage IV (metastatic) breast cancers were not included in this study. Most stage I–III breast cancer patients have surgery as the main component of their treatment [3], while for stage IV breast cancer patients, systemic therapy is the mainstay of treatment, and only some undergo surgery [15]. The NBCR combines the Auckland, Waikato, Wellington and Christchurch Registers, which includes 98% of prevalent breast cancer patients in these regions [16]. Once a patient is diagnosed with breast cancer, their information is included in this confidential register. The Waikato and Auckland Registers were collecting data prospectively before 2005 and the Christchurch and Wellington Registers started in 2009.

The NBCR includes extensive data on the demographics of these women (and men) and their tumour characteristics. This study used data on age at diagnosis, menopausal status, ethnicity, and domicile of residence, diagnosis date, mode of detection (screen-detected or symptomatic), TNM cancer stage (I, II, III and IV), grade (1, 2 and 3), biomarkers (oestrogen receptor (ER), progesterone receptor (PR) and human epidermal growth factor receptor 2 (HER2), cancer treatment including surgery, radiotherapy and systemic treatments, and whether the surgery was received in a public or a private hospital. NBCR data were linked by National Health Index (NHI) number to national-level health data, to determine diabetes status (from the VDR), comorbidities (from hospital admissions recorded in the National Minimum Dataset: NMDS) and radiotherapy (from Radiation Oncology Collection: ROC). The NHI number is a unique identifier for people receiving healthcare services in New Zealand. The VDR records diabetes patients identified using an algorithm which defines whether an individual has diabetes based on national level data on inpatient hospitalisation ICD diagnosis codes (using the NMDS), relevant outpatient events (National Non-Admitted Patient Collection, NNPAC), retinal screening (using regional retinal screening programme datasets), pharmaceutical dispensing (PHARMS) and pathology test claims (Laboratory Claims dataset). The VDR does not differentiate between types of diabetes.

Patients were classified into Māori, Pacific, Asian and European/Other ethnic groups. Ethnicity is self-identified in New Zealand. The categories of ethnicities were based on the 2018 census ethnic groups used by Statistics New Zealand [17]. Socioeconomic deprivation was defined using the New Zealand Index of Deprivation 2018 (NZDep 2018) analysed as quintile, from 1 (least deprived) to 5 (most deprived) [18]. NZ Dep is an area-level measure based on aggregated census data, not on individual data. The study period was separated into groups: 2005–2009, 2010–2014 and 2015–2020. Breast cancer subtypes were categorised into five groups according to biomarker status [19–22]: (1) luminal A: ER+ , PR+ and HER2−; (2) luminal B HER2-: ER or PR+ (but not both+), HER2−; (3) luminal B HER2+ : ER+ and/or PR+ , HER2+ ; (4) HER2 non-Luminal: ER−, PR−, HER2+ ; and (5) triple negative: ER−, PR−, HER2−. Patients ever recorded as having diabetes before cancer diagnosis in the VDR were considered to have diabetes at the time of cancer diagnosis. All comorbid conditions recorded on the NMDS for hospitalisations in the 5 years up to the index hospitalisation date were identified to calculate a C3 Index score for each patient [23]. The C3 Index is a cancer-specific index of comorbidity, with scores categorised into ‘0’ (≤ 0), ‘1’ (≤ 1.00), ‘2’ (≤ 2.00) and ‘3’ (> 2.00) [23]. Diabetes was excluded as a comorbidity from the C3 Index score calculation. Missing values in these factors were considered as a separate category to maximise the number of patients included in the data analysis.

The cancer treatments examined included BCS, mastectomy, breast reconstruction after mastectomy, and adjuvant radiotherapy after BCS. We compared the proportions of patients receiving treatments between those with diabetes and without diabetes, and examined differences between groups with; Chi-square tests. Logistic regression modelling was used to estimate the odds ratio (OR) and the 95% confidence interval (95% CI) of having no surgery (either mastectomy or BCS) for patients with diabetes compared to patients without diabetes by cancer stage, after adjustment for year of diagnosis, age, menopausal status, ethnicity, deprivation quintile, mode of detection, C3 Index score. Amongst the patients having surgery, the OR of having mastectomy in the diabetes group was estimated after adjustment for year of diagnosis, age, menopausal status, ethnicity, deprivation quintile, mode of detection, C3 score, cancer stage, grade, subtype and public/private hospital treatment. Adjusting for the same factors, we also estimated the adjusted ORs of having reconstruction after mastectomy and having radiotherapy after BCS. The OR of having surgery delay (> 31 days and > 90 days) for women with diabetes compared to women without diabetes, after adjustment for year of diagnosis, age, menopausal status, ethnicity, deprivation quintile, mode of detection, C3 score, cancer stage, grade, subtype, public/private hospital treatment and type of surgery. The ORs were estimated before and after stratification by cancer stage and ethnic group.

All analyses were performed in R Studio (Massachusetts, United States). Ethical approval for the study was granted through the University of Waikato Human Research Ethics Committee (reference: HREC(Health)2021#89).

## Results

We identified 25,557 women diagnosed with stage I–III breast cancer in 2005–2020, including 12,500 stage I, 9,807 stage II and 3250 stage III (Table 1). Of these patients, 22,651 (88.6%) had no diabetes and 2906 (11.4%) had diabetes at the time of breast cancer diagnosis. The proportion of Māori with diabetes was 19.8%, Asian 17.1% and Pacific 31.3% compared with only 8.6% in Europeans/Others. Patients with diabetes at the time of cancer diagnosis were more likely to have other comorbidities (45% had a C3 score > 0) than patients without diabetes (17.2% had a C3 score > 0). Almost one in five (560, 19.3%) patients with diabetes had a C3 Index score of 3 compared to 4.1% of patients without diabetes. The age profile for women in the diabetes group was older (mean = 65.6 years) than for women in the non-diabetes group (mean = 58.6 years).

**Table 1:** Characteristics of women with stage I-III breast cancer by diabetes status

Subgroup	No diabetes *n* (%)		Diabetes *n* (%)		*p*-value (Chi-square test)	Total *n* (%)	
Year of diagnosis							
2005–2009	4349	(19.2%)	409	(14.1%)	< 0.001	4758	(18.6%)
2010–2014	7424	(32.8%)	857	(29.5%)		8281	(32.4%)
2015–2020	10,878	(48.0%)	1640	(56.4%)		12,518	(49.0%)
Ethnicity							
Māori	2058	(9.1%)	509	(17.5%)	< 0.001	2567	(10.0%)
Pacific	1067	(4.7%)	485	(16.7%)		1552	(6.1%)
Asian	1822	(8.0%)	375	(12.9%)		2197	(8.6%)
European/others	17,704	(78.2%)	1537	(52.9%)		19,241	(75.3%)
Age (years)							
< 45	3081	(13.6%)	108	(3.7%)	< 0.001	3189	(12.5%)
45–59	9338	(41.2%)	818	(28.1%)		10,156	(39.7%)
60–69	5674	(25.0%)	944	(32.5%)		6618	(25.9%)
70–79	2724	(12.0%)	598	(20.6%)		3322	(13.0%)
80 +	1834	(8.1%)	438	(15.1%)		2272	(8.9%)
Menopausal status							
Pre-menopausal	7104	(31.4%)	358	(12.3%)	< 0.001	7462	(29.2%)
Peri-menopausal	1177	(5.2%)	74	(2.5%)		1251	(4.9%)
Post-menopausal	14,188	(62.6%)	2464	(84.8%)		16,652	(65.2%)
Unknown	182	(0.8%)	10	(0.3%)		192	(0.8%)
C3 Index score							
0	18,746	(82.8%)	1609	(55.4%)	< 0.001	20,355	(79.6%)
1	1951	(8.6%)	387	(13.3%)		2338	(9.1%)
2	1034	(4.6%)	350	(12.0%)		1384	(5.4%)
3	920	(4.1%)	560	(19.3%)		1480	(5.8%)
Deprivation quintile							
1 (least deprived)	5290	(23.4%)	373	(12.8%)	< 0.001	5663	(22.2%)
2	5036	(22.2%)	469	(16.1%)		5505	(21.5%)
3	4719	(20.8%)	556	(19.1%)		5275	(20.6%)
4	4292	(18.9%)	657	(22.6%)		4949	(19.4%)
5 (most deprived)	3144	(13.9%)	845	(29.1%)		3989	(15.6%)
Unknown	170	(0.8%)	6	(0.2%)		176	(0.7%)
Cancer stage							
Stage I	11,241	(49.6%)	1259	(43.3%)	< 0.001	12,500	(48.9%)
Stage II	8554	(37.8%)	1253	(43.1%)		9807	(38.4%)
Stage III	2856	(12.6%)	394	(13.6%)		3250	(12.7%)
Cancer grade							
1	4847	(21.4%)	518	(17.8%)	< 0.001	5365	(21.0%)
2	10,161	(44.9%)	1277	(43.9%)		11,438	(44.8%)
3	6336	(28.0%)	768	(26.4%)		7104	(27.8%)
Unknown	1307	(5.8%)	343	(11.8%)		1650	(6.5%)
Subtype							
Luminal A	13,931	(61.5%)	1872	(64.4%)	< 0.001	15,803	(61.8%)
Luminal B HER2-	2290	(10.1%)	260	(8.9%)		2550	(10.0%)
Luminal B HER2 +	2350	(10.4%)	246	(8.5%)		2596	(10.2%)
HER2 + non-luminal	948	(4.2%)	97	(3.3%)		1045	(4.1%)
Triple Negative	1899	(8.4%)	213	(7.3%)		2112	(8.3%)
Unknown	1233	(5.4%)	218	(7.5%)		1451	(5.7%)
Mode of detection							
Screen-detected	9811	(43.3%)	1234	(42.5%)	0.427	11,045	(43.2%)
Symptomatic	12,400	(54.7%)	1607	(55.3%)		14,007	(54.8%)
Unknown	440	(1.9%)	65	(2.2%)		505	(2.0%)
Health facility of surgery							
Private	6114	(27.0%)	357	(12.3%)	< 0.001	6471	(25.3%)
Public	14,111	(62.3%)	2132	(73.4%)		16,243	(63.6%)
Unknown	2426	(10.7%)	417	(14.3%)		2843	(11.1%)
Total	22,651		2906			25,557	

Most breast cancer patients (95.3%) had surgery, with 53.5% (13,672) receiving BCS and 41.8% (10,683) receiving mastectomy (Table 2). The proportion of patients not having surgery in the diabetes group (10.0%) was more than twice the proportion in the non-diabetes group (4.0%). Across stage I-III (all analysed patients), the unadjusted OR of not having surgery in women with diabetes compared to women without diabetes was 2.68 (95% CI 2.34–3.08; Table 3). After adjustment for year of diagnosis, age, menopausal status, ethnicity, deprivation quintile, mode of detection, C3 score and cancer stage, there was no substantive difference (OR 1.12, 95% CI 0.94–1.33). However, analysis for individual stages showed that for patients with stage I disease there was a higher odds of non-treatment for those with diabetes (OR: 1.45, 95% CI 1.05–2.00). The sequentially adjusted logistic regression results showed that age, ethnicity and the C3 comorbidity score had a substantial impact on the ORs of not having surgery.

**Table 2:** Surgical treatment for breast cancer patients by diabetes status

Surgery	No diabetes *n* (%)		Diabetes *n* (%)		*p*-value (Chi-square test)	Total *n* (%)	
Stage I							
Breast conserving surgery	8056	(71.7%)	889	(70.6%)	< 0.001	8945	(71.6%)
Mastectomy	2957	(26.3%)	297	(23.6%)		3254	(26.0%)
No surgery	228	(2.0%)	73	(5.8%)		301	(2.4%)
Stage II							
Breast conserving surgery	3685	(43.1%)	437	(34.9%)	< 0.001	4122	(42.0%)
Mastectomy	4326	(50.6%)	643	(51.3%)		4969	(50.7%)
No surgery	543	(6.3%)	173	(13.8%)		716	(7.3%)
Stage III							
Breast conserving surgery	534	(18.7%)	71	(18.0%)	< 0.001	605	(18.6%)
Mastectomy	2184	(76.5%)	276	(70.1%)		2460	(75.7%)
No surgery	138	(4.8%)	47	(11.9%)		185	(5.7%)
Total	22,651		2906			25,557

**Table 3:** Odds ratio of having no surgery for patients with diabetes compared to patients without diabetes by stepwise logistic regression models

Adjusted factors^a^	Stage I	Stage II	Stage III	Overall
None	2.97 (2.27–3.90)	2.36 (1.97–2.84)	2.67 (1.88–3.78)	2.68 (2.34–3.08)
Year of diagnosis	2.89 (2.20–3.79)	2.29 (1.91–2.76)	2.59 (1.82–3.68)	2.61 (2.28–3.00)
Age	2.16 (1.60–2.92)	1.63 (1.33–2.01)	1.76 (1.20–2.57)	1.85 (1.59–2.17)
Menopausal status	2.17 (1.60–2.93)	1.63 (1.32–2.01)	1.76 (1.20–2.58)	1.85 (1.59–2.17)
Ethnicity	1.94 (1.42–2.63)	1.35 (1.09–1.67)	1.50 (1.01–2.21)	1.55 (1.32–1.82)
Deprivation	1.87 (1.38–2.55)	1.33 (1.07–1.65)	1.42 (0.96–2.11)	1.51 (1.29–1.77)
Mode of detection	1.87 (1.37–2.55)	1.32 (1.07–1.64)	1.45 (0.98–2.16)	1.50 (1.28–1.76)
C3 score	1.45 (1.05–2.00)	0.98 (0.78–1.23)	1.23 (0.81–1.86)	1.14 (0.97–1.35)
Cancer stage	–	–	–	1.12 (0.94–1.33)

^a^New factor added into the model in the row above

Amongst those who had surgery, patients with diabetes were more likely to have more than 31 days delay from diagnosis to surgery, with an unadjusted OR of 1.62 (95% CI 1.49–1.76, Table 4) and an adjusted OR of 1.16 (95% CI 1.05–1.27). The difference was more substantial in women with stage I breast cancer (adjusted OR: 1.21, 95% CI 1.05–1.38). However, there was little evidence for a difference in 90 days delay of having surgery between the diabetes group and the non-diabetes group after adjustment for key sociodemographic and clinical covariates (adjusted OR 1.04, 95% CI 0.88–1.24; Table 4). A subgroup analysis looking at the different ethnic groups showed no difference in likelihood of delay by ethnicity.

**Table 4:** Odds ratio of having more than 31 and 90 days delay of having surgery for diabetes patients compared to non-diabetes

Cancer stage	Unadjusted odds ratio	Adjusted odds ratio^a^
31 days delay		
Stage I	1.66 (1.47–1.87)	1.21 (1.05–1.38)
Stage II	1.61 (1.42–1.83)	1.12 (0.96–1.30)
Stage III	1.54 (1.22–1.94)	1.17 (0.89–1.52)
Overall	1.62 (1.49–1.76)	1.16 (1.05–1.27)
90 days delay		
Stage I	0.98 (0.73–1.32)	1.11 (0.80–1.54)
Stage II	1.22 (0.97–1.54)	1.16 (0.89–1.51)
Stage III	0.84 (0.63–1.13)	0.86 (0.62–1.20)
Overall	1.07 (0.93–1.24)	1.04 (0.88–1.24)

^a^Adjusted for year of diagnosis, age, menopausal status, ethnicity, deprivation quintile, mode of detection, C3 score, cancer stage, grade, subtype, public/private hospital and type of surgery

The percentage of patients having BCS decreased over cancer stages from 71.6% for stage I disease to 18.6% for stage III disease, and the percentage having mastectomy increased from 26.0% for stage I disease to 75.7% for stage III disease. This was consistent in both diabetes and non-diabetes groups (Table 2). Before adjustment for other factors, women with diabetes were more likely to have mastectomy instead of BCS than women without diabetes (OR 1.13, 95% CI 1.04–1.22, Table 5). However, the use of mastectomy between the two groups was substantively similar after adjustment for age, menopausal status, ethnicity, deprivation quintile, year of diagnosis, mode of detection, C3 score, cancer stage, grade and subtype (OR 0.98, 95% CI 0.89–1.08).

**Table 5:** Odds ratio of having mastectomy versus BCS for patients with diabetes compared to patients without

Cancer stage	Unadjusted odds ratio	Adjusted odds ratio^a^
Stage I	0.91 (0.79–1.04)	0.88 (0.75–1.02)
Stage II	1.25 (1.10–1.43)	1.13 (0.98–1.30)
Stage III	0.95 (0.72–1.25)	0.82 (0.60–1.11)
Overall	1.13 (1.04–1.22)	0.98 (0.89–1.08)

^a^Adjusted for year of diagnosis, age, menopausal status, ethnicity, deprivation quintile, mode of detection, C3 score, cancer stage, grade, subtype and public/private hospital

Breast reconstruction surgery was examined amongst those who has mastectomy. Amongst women without diabetes who had mastectomy, 2362/9467 (24.9%) had breast reconstruction compared to 68/1216 (5.6%) of women with diabetes (Appendix Table 7). This patterning was apparent within each cancer stage group, though the proportion of women having reconstruction after mastectomy decreased with cancer stage for both the diabetes and non-diabetes group, from 9.4% for stage I cancer to 3.3% for stage III cancer in the diabetes group, and from 32.4% for stage I cancer to 18.2% for stage III cancer in the non-diabetes group. The unadjusted OR for reconstruction after mastectomy for women with diabetes compared to women without diabetes decreased with cancer stage, from 0.22 (95% CI 0.15–0.32) for stage I cancer, to 0.16 (95% CI 0.11–0.24) for stage II and 0.15 (95% CI 0.08–0.30) for stage III (Table 6). After adjustment for year of diagnosis, age, menopausal status, ethnicity, deprivation quintile, mode of detection, C3 score, cancer stage, grade, subtype and public/private hospital, the same pattern remained but the difference between those with diabetes compared to those no diabetes was attenuated: from 0.54 (95% CI 0.35–0.84) for stage I cancer to 0.50 (95% CI 0.34–0.75) for stage II and 0.48 (95% CI 0.24–1.00) for stage III cancer.

**Table 6:** Odds ratio of having reconstruction after mastectomy and having radiotherapy after BCS for diabetes patients compared to non-diabetes

Cancer stage	Unadjusted odds ratio	Adjusted odds ratio^a^
Reconstruction after mastectomy		
Stage I	0.22 (0.15–0.32)	0.54 (0.35–0.84)
Stage II	0.16 (0.11–0.24)	0.50 (0.34–0.75)
Stage III	0.15 (0.08–0.30)	0.48 (0.24–1.00)
Overall	0.18 (0.14–0.23)	0.51 (0.39–0.67)
Radiotherapy after BCS		
Stage I	0.83 (0.70–0.99)	1.01 (0.83–1.22)
Stage II	0.74 (0.57–0.95)	0.99 (0.74–1.32)
Stage III	0.78 (0.36–1.65)	1.34 (0.56–3.17)
Overall	0.81 (0.70–0.93)	1.01 (0.86–1.18)

^a^Adjusted for year of diagnosis, age, menopausal status, ethnicity, deprivation quintile, mode of detection, C3 score, cancer stage, grade, subtype and public/private hospital

Receipt of radiotherapy after BCS increased with cancer stage for both diabetes and non-diabetes groups, from 79.6% and 82.4% for stage I cancer to 87.3% and 89.9% for stage III cancer, respectively (Appendix Table 7). Before adjustment for other factors, women with diabetes were less likely to have radiotherapy after BCS than women without diabetes, (OR 0.81, 95% CI 0.70–0.93) for stage I–III cancers (Table 6). However, adjustment for other factors largely explained this difference (adjusted OR 1.01, 95% CI 0.86–1.18). The ORs of different treatments by ethnic group have similar patterns, though the respective ORs can be slightly different by ethnic group (Appendix Table 8).

**Table 7:** Proportion of patients having reconstruction after mastectomy and having radiotherapy after BCS between patients with diabetes and patients without

Cancer stage	No diabetes *n* (%)		Diabetes *n* (%)		*p*-value (Chi-square test)	Total *n* (%)	
Reconstruction after mastectomy							
Stage I							
No reconstruction	1999	(67.6%)	269	(90.6%)	< 0.001	2268	(69.7%)
Reconstruction	958	(32.4%)	28	(9.4%)		986	(30.3%)
Stage II							
No reconstruction	3309	(76.5%)	612	(95.2%)	< 0.001	3921	(78.9%)
Reconstruction	1017	(23.5%)	31	(4.8%)		1048	(21.1%)
Stage III							
No reconstruction	1787	(81.8%)	267	(96.7%)	< 0.001	2054	(83.5%)
Reconstruction	397	(18.2%)	9	(3.3%)		406	(16.5%)
Radiotherapy after BCS							
Stage I							
No adjuvant radiotherapy	1416	(17.6%)	181	(20.4%)	0.040	1597	(17.9%)
Adjuvant radiotherapy	6640	(82.4%)	708	(79.6%)		7348	(82.1%)
Stage II							
No adjuvant radiotherapy	565	(15.3%)	86	(19.7%)	0.018	651	(15.8%)
Adjuvant radiotherapy	3120	(84.7%)	351	(80.3%)		3471	(84.2%)
Stage III							
No adjuvant radiotherapy	54	(10.1%)	9	(12.7%)	0.506	63	(10.4%)
Adjuvant radiotherapy	480	(89.9%)	62	(87.3%)		542	(89.6%)

**Table 8:** Odds ratio of having surgery vs no surgery for diabetes patients compared to non-diabetes by ethnic group

Treatment	Māori	Pacific	Asian	European/others
Surgery	1.33 (0.83–2.13)	1.15 (0.72–1.83)	1.24 (0.59–2.59)	1.08 (0.88–1.33)
> 31 days delay for surgery	1.14 (0.89–1.45)	0.96 (0.73–1.28)	1.29 (0.98–1.68)	1.15 (1.02–1.30)
> 90 days delay for surgery	1.06 (0.70–1.60)	0.89 (0.59–1.35)	1.42 (0.81–2.47)	1.01 (0.79–1.28)
Mastectomy vs BCS	1.09 (0.87–1.41)	0.93 (0.71–1.26)	0.91 (0.69–1.21)	0.93 (0.82–1.06)
Reconstruction after mastectomy	0.41 (0.19–0.91)	0.38 (0.13–1.11)	0.78 (0.41–1.47)	0.51 (0.35–0.73)
Radiotherapy after BCS	0.98 (0.65–1.46)	0.95 (0.60–1.50)	0.83 (0.49–1.38)	1.03 (0.84–1.27)

Same adjusted factors as the analyses by cancer stage

## Discussion

Women diagnosed with localised (Stage I–III) breast cancer face a number of decisions about their treatment. Firstly, whether to have surgery to remove the tumour. There is then the decision whether to have a BCS or a mastectomy, and if mastectomy is the preferred option whether to have a breast reconstruction. All these decisions are taken in light of the individual’s general wellbeing, age and personal choice. Women with both diabetes and breast cancer in this study tended to be older, were more likely to be Māori, Pacific or Asian, to be post-menopausal, to have more comorbidities, to be more deprived, have more advanced disease and to have more luminal A disease. The additional risks of surgery and anaesthetic complications associated with having diabetes need to be taken into account when surgical treatment choices are made. Patients should be involved in these decisions, but it is not possible when using an observational database to know whether these choices are patient or provider driven.

This study shows the impact diabetes has on treatment for breast cancer. The effects on surgical treatment vary by cancer stage and surgery type. The probability of not having surgery for women with diabetes at the time of breast cancer diagnosis was more than twice that of women without diabetes at cancer diagnosis. Age, ethnicity and comorbidities are the key contributors to these differences. As noted above women who had diabetes were more likely to be older, be Māori, Pacific or Asian and have more comorbidities than women without diabetes. Previous studies have shown that older age and more comorbidities are significantly associated with decreasing use of surgery [3, 24–26]. Therefore, after adjustment for age, comorbidities and other factors, the gap in use of surgery for breast cancer between the diabetes group and the non-diabetes group narrowed, and the difference was only significant for women with stage I breast cancer. Important comorbidities (especially poor cardiac or lung function), are a contraindication to undergoing breast cancer surgery [26], and diabetes is an added challenge, particularly raising the risk of postoperative infection, as well as being associated with cardiovascular disease, renal impairment and obesity.

Women with diabetes at cancer diagnosis were less likely to have breast reconstruction after mastectomy than women without diabetes. Compared to BCS and mastectomy which are curative surgeries, breast reconstruction is associated with better body image, sexuality and self-esteem, as well as lower rates of depression [27]. Only 22.8% of women with stage I-III breast cancer had reconstruction after mastectomy. An important factor in the decision to decline breast reconstruction is the age of the patients [28]. Older age, more comorbidities and higher Body Mass Index (BMI) in the diabetes group explained the differences in use of breast reconstruction between the diabetes group and the non-diabetes group. One-year increase in age is associated with an OR of 0.91 (0.90–0.92) decrease likelihood of having breast reconstruction, and women with two or more comorbidities are less than half as likely to undergo reconstruction than women without comorbidities [3]. Even after adjustment for age and other comorbidities, the presence of diabetes still has a substantial impact on the use of breast reconstruction. The Breast Reconstruction Expert Advisory Group supports delivery of breast reconstruction up to a BMI of 35 [29]. Performing breast reconstruction on patients with a high BMI can lead to a high complication rate [29]. We also know that Māori are less likely to receive breast reconstruction after mastectomy [3, 4]. However, a subgroup analysis by ethnicity (Appendix Table 8) showed no difference in the use of breast reconstruction between those with and without diabetes, suggesting that having diabetes rather than a patient’s ethnicity is the main driver in this decision-making.

Women with diabetes were less likely to receive radiotherapy after BCS (OR 0.81) than women without diabetes, but the differences were no longer apparent after adjustment for other comorbidities and other factors. This is similar to findings from in a Canadian study of 4955 women with breast cancer and diabetes and 9910 matched control patients with breast cancer but without diabetes [10]. This study demonstrated a relative risk of 0.97 (95% CI 0.95–0.99) of having radiotherapy after BCS for women with diabetes compared to women without diabetes. After adjustment for cardiovascular disease, renal disease, dementia, and ADG score, the difference became not apparent in the rate of receipt of radiotherapy between women with diabetes who had BCS and those without diabetes [10]. This suggests that diabetes alone does not impact on the use of radiotherapy in women who have had BCS.

Treatment delay can adversely affect patient outcomes. Women with diabetes at cancer diagnosis were more likely to have greater than 31 days delay in having breast cancer surgery than women without diabetes. This is consistent with an earlier New Zealand study which demonstrated that comorbidity including diabetes is associated with a higher risk of having treatment delay after adjustment for ethnicity, surgery type, public/private hospital and other factors [30]. It is notable that there was no strong evidence for a differential delay after 90 days from diagnosis and so the clinical impact of the finding of delay may be minimal. Before adjustment for comorbidities and other factors, the diabetes group were more likely to have mastectomy instead of BCS than the non-diabetes group. However, the differences are no longer important after adjustment. This is consistent with other studies which showed that women who are older and have more comorbidities were more likely to have mastectomy instead of BCS than others [3, 31].

The strengths of this study include utilisation of the comprehensive NBCR recording detailed data on patient treatments including type of treatment and treatment date, and the use of a national prevalent diabetes database to establish diabetes status. This enabled us to examine the association of diabetes with breast cancer treatments and treatment delay. These breast cancer data were linked to other health data to allow for adjustment for the impact of comorbidities when estimating the impact of diabetes on breast cancer treatment. This study also has limitations. The VDR dataset does not differentiate between type 1 and type 2 diabetes, therefore we could not examine the differences between type 1 diabetes and type 2 diabetes on the impact of cancer treatment. As with most studies using existing data, we could not explore the reasons driving differences in treatment between the diabetes and non-diabetes group. For instance, a small proportion of women with localised breast cancer will receive neoadjuvant therapy and could be a confounding factor but these data were not available for inclusion in our modelling.

## Conclusions

Diabetes has an impact on the surgical treatment of New Zealand women with breast cancer. Women with Stage I–III breast cancer and diabetes are less likely to undergo surgery but much of this difference was accounted for differences in age and the presence of other comorbidities of older patients with diabetes. Ethnicity also seems to be a confounding variable that is both associated with the presence of diabetes and the likelihood of not receiving surgery. Diabetes also seems to lower the likelihood of having breast reconstruction after mastectomy and leads to initial treatment delay. These differences need to be taken in to account when considering factors that may impact on overall outcomes for women with breast cancer and diabetes. This is especially true for those ethnic groups who have a high prevalence of diabetes and who have poorer outcomes from breast cancer such as Māori and Pacific women.

## Data Availability

The data used for this study are not publicly available because of the ethics for patient information. They can be access through the National Breast Cancer Register and the Ministry of Health with appropriate ethics approval.

## Appendix

See Tables 7 and 8
